# 

**Published:** 1898-03-26

**Authors:** 


**Notices of Births, Deaths, and Marriages are inserted in this column at a charge of 2s. 6d. for an announcement not exceeding four lines.**

### NOTICE TO CORRESPONDENTS.

**All MS., Letters, Books for Review, and other matters intended for the Editor should be addressed The Editor, "The Hospital," The Hospital Building, 28 & 29, Southampton Street, Strand, W.C.**

**The Editor cannot undertake to return rejected MS., even when accompanied by stamped directed envelope.**

**Notice to Advertisers and Subscribers.**

**All Advertisements, Orders for Copies of The Hospital, and Business Communications should be addressed to THE MANAGER, (Not to The Editor), The Hospital, The Hospital Building, 28 & 29, Southampton Street, Strand, London, W.C.**

**The Cost of Subscription, post free, is as follows:—Per week, 2½d. per quarter, 2s. 8d.; per half-year, 5s. 3d.; per annum, 10s. 6d. Foreign:—Per annum, 15s. 2d.; payable, in all cases, in advance.**

**NOTICE.—Vol. XXII. of "The Hospital" (April, 1897, to September, 1897), in handsome cloth binding, is now on sale at the Office of this Journal, price 7s. 6d. Cases for binding the Numbers contained in this Volume, 1s. 6d. Index to Vol. XXII., 2½d., post free. The Monthly Part for April will be ready on April 1st. Price 6d., by post 8d.**

### BOOKS RECEIVED.

Queensland Agent-General's Office.

"Ethnological Studies Among the Queensland Aborigines."

H. K. Lewis.

"Diseases of the Nervous System." By Charles E. Beevor, M.D., F.R.C.P.

"Cleveland Medical Gazette" Publishing Co.

"About Children." By Samuel W. Kelly, M.D.

The Savoy Press.

"Stammering and Stuttering, and Other Speech Affections." By W. Abbotts, M.D.

John Wright and Co.

"Doctor and Patient." By Dr. Robert Guernsey, with a Preface by D. J. Leech, M.D.

Simpkin Marshall, Hamilton, Kent, and Co.

"The Angel Hermit." By A. W. Letts.

Arthur Pearson.

"A Healthy Home." By Dr. Andrew Wilson.

John Long.

"Trewinnot of Guy's." By Mrs. Coulson Kernahan.

J. W. Arrowsmith.

"Some Incidents in General Practice." By Augustin Pritchard.

**Periodicals and Pamphlets.**—The Sun, The Critic, Knowledge, Electrical Review, Medical Record, Literary Digest, Industries and Iron, Medical Press, Asylum News, Woman's Signal.

### The Student of Medicine.

### APPOINTMENTS.

W. C. Beatley, M.D.Durh., M.R.C.S., appointed Honorary Physician-Accoucheur to the Newcastle-on-Tyne Lying-in Hospital. E. H. Betts, M.R.C.S., L.R.C.P.Lond., appointed House Surgeon to the Dorset County Hospital. Dr. Brownridge, appointed Physician to the Paisley Infirmary Dispensary. A. Burgess, M.D., appointed Medical Officer for the No. 1 District of the Lincoln Union. G. Cole, M.R.C.S.Eng., L.R.C.P.Lond., appointed Medical Officer for the Sixth District of the Nottingham Union. H. C. Darby, L.R.C.P., L.R.C.S.Edin., appointed Medical Officer of Health to the Lye and Wollescote Urban District Council. H. C. Dent, M.R.C.S.Eng., L.S.A., appointed Medical Officer for the Northrepps District of the Erpingham Union. W. Fairbanks, M.D.Edin., M.B., C.M., appointed Medical Officer to the Workhouse of the Wells Union. W. H. S. Fosbery, M.A., M.D., B.C.Cantab., appointed Medical Officer to the Reading Dispensary. E. J. Fox, B.Sc.Lond., M.R.C.S., L.R.C.P., appointed Jnnior House Surgeon to the Salford Royal Hospital. J. D. Gilruth, M.B., C.M.Edin., appointed Surgeon to the Arbroath Infirmary. Dr. Gooding, appointed Medical Officer of Health to the Barnstaple Port Sanitary Authority. Dr. Hickman, appointed Medical Officer for the Coleford District of the Frome Union. Dr. Hope, appointed Medical Officer of Health to the Hanwell Urban District Council. Dr. House, appointed Medical Officer for the No. 2 District of the Lincoln Union. F. Jaffrey, F.R.C.S.Eng., appointed Surgeon to Out-patients at the Belgrave Hospital for Children. B. Macfarland, M.D., R.U.I., M.Ch., appointed Medical Officer for the Workhouse of the Lincoln Union. Dr. Moloney, appointed Medical Officer for the Murroe Dispensary District. C. B. T. Musgrave, M.D.Lond., reappointed Medical Officer for the Cromer District of the Erpingham Union. G. P. Newbolt, M.B.Durh., F.R.C.S.Eng., appointed an Honorary Surgeon to the Royal Southern Hospital, Liverpool. J. H. Patterson, M.B., C.M.Edin., appointed House Surgeon to the Bradford Children's Hospital. W. R. Pirie, M.A., M.B, C.M.Aberd., appointed Medical Officer to the Aberdeen General Dispensary. A. M. Saunders, M.A., M.B., C.M., D.P.H.Aberd., appointed Medical Officer of Health for the Burgh of Burghead, Morayshire, N.B. W. J. Simpson, M.D.Aberd., D.P.H.Camb, appointed Professor of Hygiene at King's College, London. F. G. Smith, L.R.C.P., L.R.C.S., appointed Medical Officer for the Workhouse and the Stockton District of the Stockton Union. A. M. Watts, M.R.C.S.Eng., L.R.C.P.Lond., appointed Medical Officer for the East Ashford Union.

